# Characterization of Degradation Progressive in Composite Laminates Subjected to Thermal Fatigue and Moisture Diffusion by Lamb Waves

**DOI:** 10.3390/s16020260

**Published:** 2016-02-19

**Authors:** Weibin Li, Chunguang Xu, Younho Cho

**Affiliations:** 1 School of Aerospace Engineering, Xiamen University, Xiamen 361005, China; 2 School of Mechanical Engineering, Beijing Institute of Technology, Beijing 100041, China; xucg@bit.edu.cn; 3 School of Mechanical Engineering, Pusan National University, Busan 609-735, Korea

**Keywords:** composite laminates, degradation progressive, thermal fatigue, moisture diffusion, Lamb waves

## Abstract

Laminate composites which are widely used in the aeronautical industry, are usually subjected to frequency variation of environmental temperature and excessive humidity in the in-service environment. The thermal fatigue and moisture absorption in composites may induce material degradation. There is a demand to investigate the coupling damages mechanism and characterize the degradation evolution of composite laminates for the particular application. In this paper, the degradation evolution in unidirectional carbon/epoxy composite laminates subjected to thermal fatigue and moisture absorption is characterized by Lamb waves. The decrease rate of Lamb wave velocity is used to track the degradation evolution in the specimens. The results show that there are two stages for the progressive degradation of composites under the coupling effect of thermal cyclic loading and moisture diffusion. The present work provides an alternative to monitoring the degradation evolution of in-service aircraft composite Laminates.

## 1. Introduction

Composite laminates are finding increased applications in aircraft industries due to their advantages over conventional metallic materials, such as higher strength/weight ratio, and greater stiffness/weight ratio [1]. However, such applications of composite laminates in aircraft industries generally involve subjecting materials to the in-service environment, with the variation of temperature and excessive humidity conditions. Damage mechanism in composite structures is usually much more complex than that in isotropic solid. In particular, in-service degradation evolution of composites properties in aircraft industry is generally induced by the coupling of multi-loadings [2].

Material properties of composite laminates are largely determined by the matrix and interfacial properties, specimen geometry as well as the anisotropy. The influence of the laminate thickness, stacking sequence and thermal aging on the properties and behavior in carbon/epoxy laminates was studied by Mlyniec, *et al.* [3]. The effects of moisture on composite laminates are the formation of residual hygral compressive stresses, polymer and interfacial degradation and polymer plasticization [4]. Combinations of these effects can significantly degrade the toughness and strength of composites. The changes in mechanical and viscoelastic properties of composite materials subjected to different ageing constraints was studied in [5]. Korta, *et al.* reported the experimental and numerical study on the effect of humidity-temperature cycling on adhesive joints [6]. Recently, Mlyniec, *et al.* proposed a chemomechanical model to study the influence of elevated temperature aging and humidity-temperature cycling on epoxy adhesives, the validation of the model was also proved by the experimental investigation [7]. The progressive of damage in carbon/epoxy laminates subjected to isothermal ageing or thermal cycling was investigated in [8,9], during thermal cycling, thermal stresses appear in the laminates, which may induce transverse matrix cracks, debonding and delaminations. Even though temperature variations and moisture diffusion are known to affect fiber/matrix stress distributions and matrix properties, the degradation evolution induced by the effects of such conditions on the composites, is still not understood clearly. Tracking the material degradation evolution is a critical aspect to study the damage mechanism [10]. Therefore, there is a demand to characterize the degradation evolution during in-service aging of composite structures for maintenance of aircraft.

The use of ultrasonic Lamb waves offers a convenient method for material characterization [11]. Lamb waves can propagate over considerable distances without the need to scan point by point, and thus hidden and inaccessible area of the structure can be examined quickly. The entire thickness of the laminate can also be interrogated by appropriate mode selection and frequency turning [12]. So the use of Lamb waves provides a potential of detecting internal defects as well as that on surface [13]. Low energy consumption and great cost-effectiveness are features for Lamb wave-based damage detection approach [14]. However, unlike bulk waves, the features of Lamb waves propagation are much more complex for the multi-modes and dispersive nature. Features of Lamb waves propagation in composites have been extensively studied [15,16,17]. The exploration of Lamb wave applied in composite laminates has got increasingly concern for the reason that laminate-structures are usually relatively small thickness compared with length. Lamb waves have been applied to detecting and evaluating impact damages, delaminations, fatigue and thermal fatigue in composite laminates [18,19,20,21,22,23]. Various Lamb waves based techniques have been developed for inspecting and monitoring the damages in composites. For instance, reflection signals of Lamb wave propagation were used as an effective tool for detecting the presence and location of delaminations in composites [24], but the technique can not determine the extent of the damage with reasonable precision. The use of Lamb wave magnitude or amplitude has also been suggested as a potential way to identify defects in composites [25], the drawback of this method is that the variability in the coupling and the sensitivity of transducers could induce changes in the amplitude or the energy of the signal that are often comparable with those caused by defects. The velocimetric method, which is used to track changes in the ultrasonic velocities due to variations in material properties, can be used reliably for damage identification [26]. Variation of Lamb wave velocity was taken as an indicator of degradation induced by long-term thermo-oxidative ageing of carbon/epoxy laminates [27].

It has been shown that material properties degradation can significantly affect Lamb waves propagation in the composites. However, earlier studies mainly focus on evaluating and detecting the discontinuous in the material, there is rare investigations about tracking nondestructively the progressive degradation stages to study the coupling damages mechanism of in-service composite structures. In this paper, material degradation evolution induced by the coupling in-service damages including thermal fatigue and moisture in carbon/epoxy composites is characterized by Lamb waves. Lamb wave mode selection and experimental verification are conducted by wavelet analysis. Artificial thermal fatigue cyclic loading and water absorption are introduced into the tested specimens to simulate the effect of in-service environmental conditions on the composite structures. The correlation is presented between variation of Lamb wave velocity and material aging period. The change rate of Lamb wave velocity is used to indicate the degradation evolution in the specimens.

## 2. Lamb Waves Propagation in Composites

Lamb waves propagation in composite structures with anisotropic properties has some interesting and unique features, such as direction-dependent speed, difference between phase and group velocity. Lamb waves in a multi-layered composite laminate can be generally described by its displacement. The displacement field is governed by Navier’s equation within each layer [28,29].

(1) $$(\lambda \;+\;2\mu )\nabla (\nabla \cdot u^{n})-\mu \nabla \;\times \;(\nabla \;\times \;u^{n})\;=\;\rho_{0}\frac{\partial^{2}u^{n}}{\partial t^{2}}(n\;=\;1,2,.....N)$$

where u is the displacement, λ and μ are the elastic constants, $\rho_{0}$ is the density of the layer, respectively. While applying the traction-free boundary conditions on the surface to Equation (1), the comprehensive dispersion equation can be derived as following,

(2) $$\left|A(f,k,\lambda^{n},\mu^{n},h_{n})\right|=0$$

where f is the frequency, k is wave number and plate geometry $h_{n}$, for a given material ($\lambda^{n},\mu^{n}$). The propagating Lamb wave modes can be represented in dispersion curves through solving dispersion equations. Lamb waves in a multi-layered structure are affected by the thickness, density, and material elastic properties in each layer of the structure. The dispersion equations for guided waves in the composites satisfying traction-free boundary conditions on the two surfaces of the plate are obtained from the matrix method in the explicit form [28],

(3) $$G(f,c_{p})=0$$

where $c_{p}$ is the phase velocity of the pure guided waves propagating through the surfaces of specimen. The dispersion curves are obtained by solving the equations for $c_{p}$. Thus, for the traction free case, the dispersion function $G(f,c_{p})$ can be chosen as the function to be minimized for optimum choice of $c_{p}$ or f consistent with a selected set of data [11]. It is shown that material properties are highly affected the wave velocity of Lamb wave propagation in composites.

**Figure 1:**
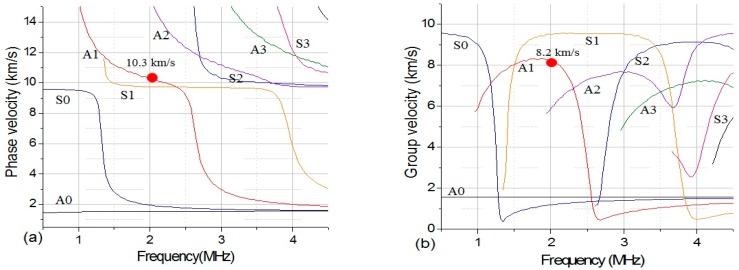
Numerically calculated phase velocity (**a**) and group velocity (**b**) dispersion curves for Lamb wave in the [0]_6_ carbon/epoxy laminates, the propagation direction of the waves is along the fiber direction.

The dispersion curves of Lamb waves propagation in the specimens used in the present investigation were calculated numerically using the classical transfer matrix method for layered media. As shown in Figure 1, the A1 mode at frequency 2 MHz with phase velocity 10.3 km/s is chosen in the present investigation. Piezoelectric transducers (PZT) and wedges are used in this work for the generation and detection of the Lamb waves. The angle of incidence for generating Lamb wave modes is determined by Snell’s law. In experimental work, when we generated the A1 Lamb wave mode as shown in Figure 1a, it is inevitable that S1 mode will also be generated. However, as shown in Figure 1b, the group velocity of S1 mode is quite different from that of A1 mode. So, after a certain propagation distance, wave-packs of S1 and A1 wave mode will eventually separate. In this approach, it is possible to selectively choose only the A1 mode part. The detailed introduction of the group delay approach was also reported in [9,19].

**Figure 2:**
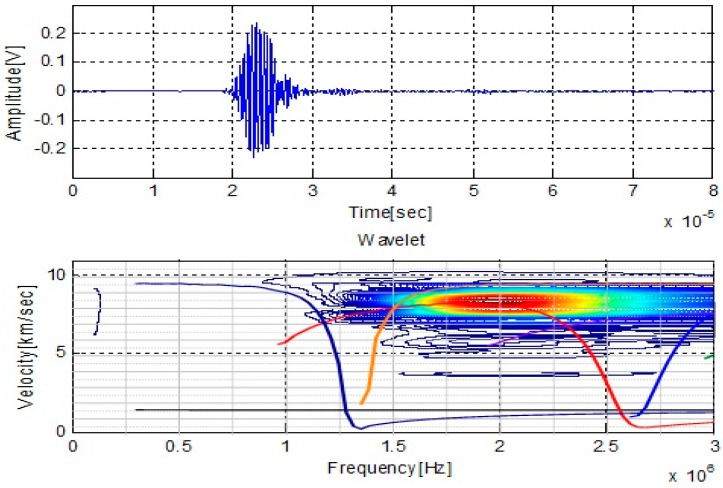
Wavelet analysis of experimental signal for mode verification.

As we discussed in above, the dispersive and multi-modes nature of guided waves make the experimental test complicated. It is essential to verify that the incident Lamb wave mode is the expected signal. To verify the experimental signal, the wavelet transform is used to check the frequency spectrum and group velocity of the signal in this investigation. Wavelet is an effective tool for the experimental analysis of the dispersive waves in the composites [29,30]. The arrival times of each frequency component needed in the velocity calculation could be determined from the peak of the magnitude of wavelet data on the time-frequency plane. A time-frequency analysis using wavelet transform was performed firstly to verify the Lamb wave modes that present on the signal acquired form the specimen without damage. As can be seen from time-frequency analysis in Figure 2, the arrival times of group velocity at each frequency of the signal can be extracted by using the peak of the magnitude. The group velocity of signal is close to 8.2 km/s, and the central frequency of the experimental signal is 2 MHz. Comparing the values of experimental signal with those shown in numerical dispersion curve, it can be verified that the propagating signal is the expected A1 wave mode.

## 3. Specimens and Experimental Setup

**Figure 3:**
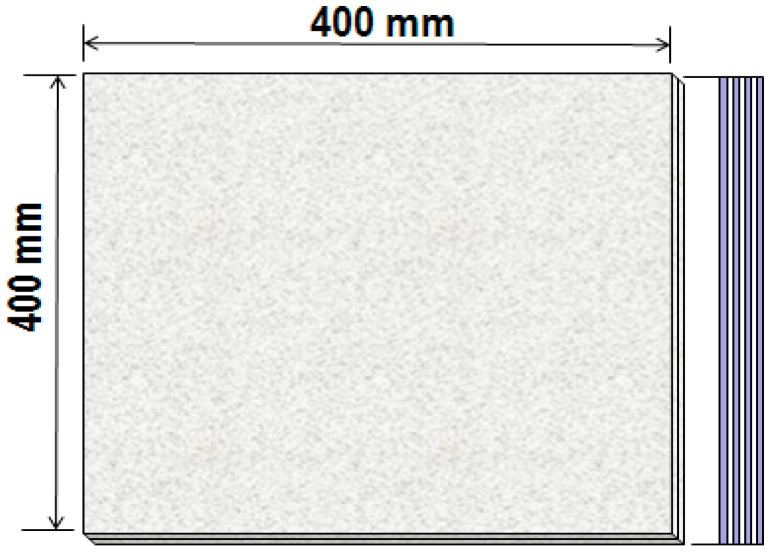
Shape of tested samples.

All experimental tests were carried out in unidirectional carbon/epoxy laminates. The tested laminates are made of 6 plies with 1.0 mm thickness. The dimensions are 400 mm × 400 mm as shown in Figure 3. All three tested specimens have the same dimensions and provided by the same supplier. Table 1 shows the density and elastic stiffness coefficients of the material. Before testing, the samples have been dried to reach a dry state of reference.

**Figure 4:**
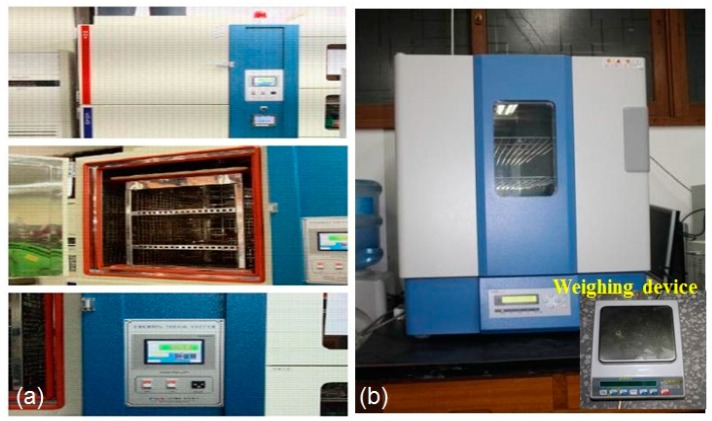
Instruments of temperature-humidity chamber (**a**) for thermal fatigue (**b**) for moisture diffusion.

Thermal fatigue was firstly imposed on the specimens to cause thermal degradation. The maximum and minimum temperatures of thermal cycle were 70 °C and −55 °C, respectively, with a constant cooling and heating time of 15 min. Two of the specimens in this study were subjected to 1000, 2000 thermal fatigue cycles, respectively. The instruments used to introduce thermal fatigue and moisture absorption into specimens are shown in Figure 4.

After thermal fatigue cycling, ultrasonic Lamb wave tests were conducted on the three specimens. Prior to moisture absorption, specimens were dried in an oven for 24 h at 120 °C. Weight gain was monitored by periodically removing from the chamber. Lamb wave velocity variation and weights recording were examined in the specimens under different moisturizing stages. Water absorption of specimens follows the Fickian phenomenon in the case of used materials [31,32]. There is a quite good correlation between absorption and square root of time. So, the percentage of moisture diffusion in the specimens can be approximately controlled by the moisturizing time. In the current investigation, 24 h moisture absorption was taken as one stage. In total, six stages were imposed on the three different specimens subjected to various thermal fatigue loadings, as shown in Table 2. The quantity of moisture diffusion in the composites is indicated by the weight of water absorption in specimens. Moisture content is illustrated as the percent weight gain of the material as,

(4) $$M\;=\;\frac{W_{\left(m\right)}-W_{\left(d\right)}}{W_{\left(d\right)}}\;\times \;100$$

where, $W_{\left(m\right)}$ is weight of moist material. $W_{\left(d\right)}$ is weight of dry material.

**Figure 5:**
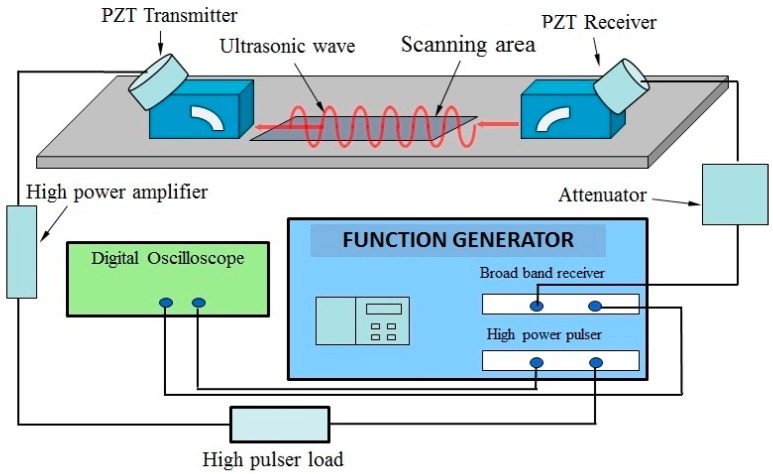
Ultrasonic measurement system.

The experimental setup to monitor the variation of ultrasonic wave velocity is shown in Figure 5. A high-power tone burst signal of 10 cycles at frequency of 2 MHz is generated by the RAM-RITEC ultrasonic measurement system. More detailed introduction of this ultrasonic measurement system can be found in [33]. The transmitter and receiver unit are equipped with a high power attenuator and a high power 50 Ω amplifier. Ultrasonic angle changeable transducer with central frequency of 2 MHz is used to generate the Lamb wave mode. A similar angle changeable transducer with central frequency of 2 MHz is chosen to receive the propagating wave signal. A fixed pressure is loaded in the transducers through holders. The transducers are coupled to the specimen with light lubrication oil.

## 4. Results and Discussions

During thermal cycling, thermal stresses appear in the composite laminates due to the difference in the coefficient of thermal expansion (CTE) between the fibers and the matrix [34]. These cyclic thermal stresses may induce transverse matrix cracks, debonding and delaminations [35]. The matrix cracks induced by cyclic thermal stress offer to the process of oxidation many access paths towards the interior of the material. When these cyclic temperature variations occur in an oxidative environment, another kind of damage, dependent on the matrix oxidation, appears. Composite oxidation results in reduction in volume of the resin inducing shrinkage of the matrix relatively to fibers [9,34]. The effects of moisture on composite laminates are the formation of residual hygral compressive stresses, polymer and interfacial degradation and polymer plasticization. Combinations of these effects can significantly degrade the toughness and strength of composites [36]. The velocity of Lamb waves in the composites is highly depended on the material stiffness matrix [37]. The decrease of Lamb wave velocity is attributed to the degradation of the composite properties. The higher wave velocity corresponds to the better material properties of waveguide [19,38]. From this view point, we can predict the material degraded state by checking the variation of Lamb wave velocity in the specimens.

**Figure 6:**
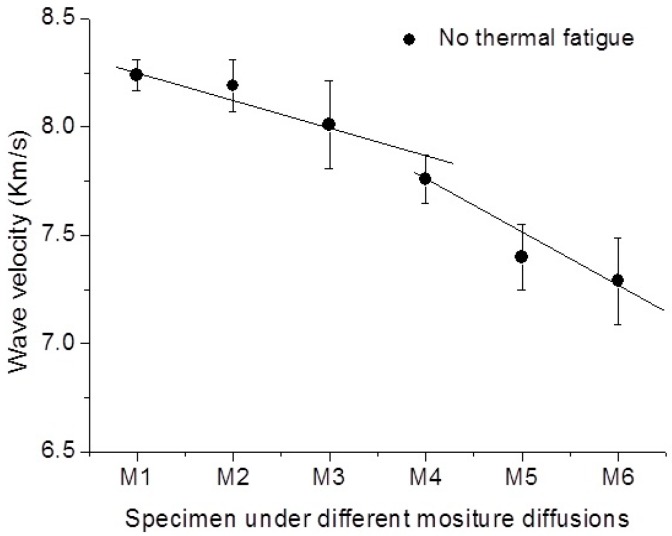
Variation of wave velocity in the specimen, which subjected no thermal fatigue, in different moisture diffusional states.

The variation of Lamb wave velocity in the specimen that only suffered the moisture diffusion is presented in Figure 6. It shows that the decrease rate of wave velocity is slow with respect to the increase of moisture diffusion. There is no oscillation of wave velocity variation in this specimen, which indicates a relative constant manner of degradation progressive in composites under the pure moisture absorption.

**Figure 7:**
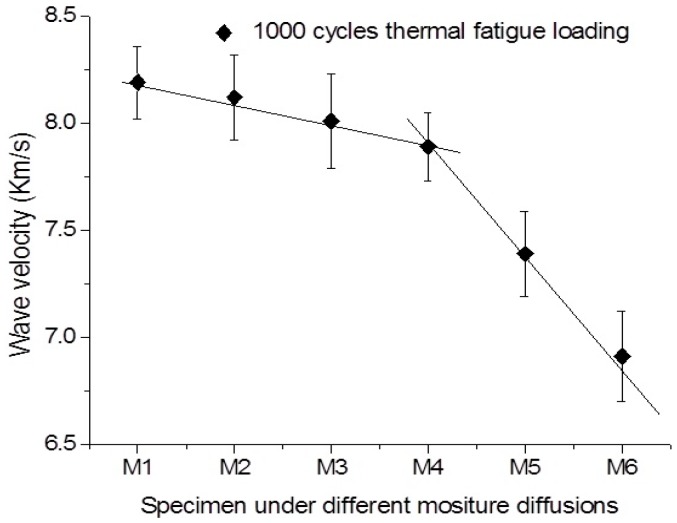
Variation of wave velocity in the specimen, which subjected 1000 cycles thermal fatigue, in different moisture diffusional states.

**Figure 8:**
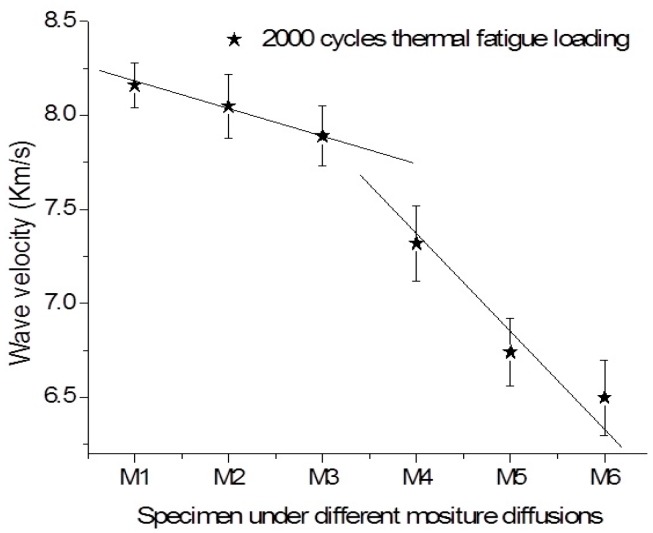
Variation of wave velocity in the specimen, which subjected 2000 cycles thermal fatigue, in different moisture diffusional states.

However, as shown in Figure 7 and Figure 8, the variations of wave velocity in the specimens subjected to thermal fatigue damages at different moist states. With the increase of moisture diffusion in the specimen, the sharply decrease of wave velocity in the specimen subjected thermal fatigue is happened. It is found that the slopes of line representing the degradation progressive is higher that of the sample without suffering thermal damage. It can be observed that the decrease rate of wave velocity in the virgin sample is also lower compared to that of the samples under multi-damages. Interestingly, the sharply change of the Lamb wave velocity in the specimen subjected to 1000 thermal cycles is not as earlier as that in specimen subjected 2000 thermal cycles, as the increase of moisturizing stages. The decrease rates of wave velocity at the initial moisturizing stages are almost same for the both cases. It can be concluded that the variations of Lamb wave velocity induced by material degradation are mainly attributed to moisture at the initial stage. The apparent increase of matrix cracks, as well as fiber/matrix debondings caused by the increase of thermal cycles will only accelerate the water absorption and degrading the material properties in later stage.

**Figure 9:**
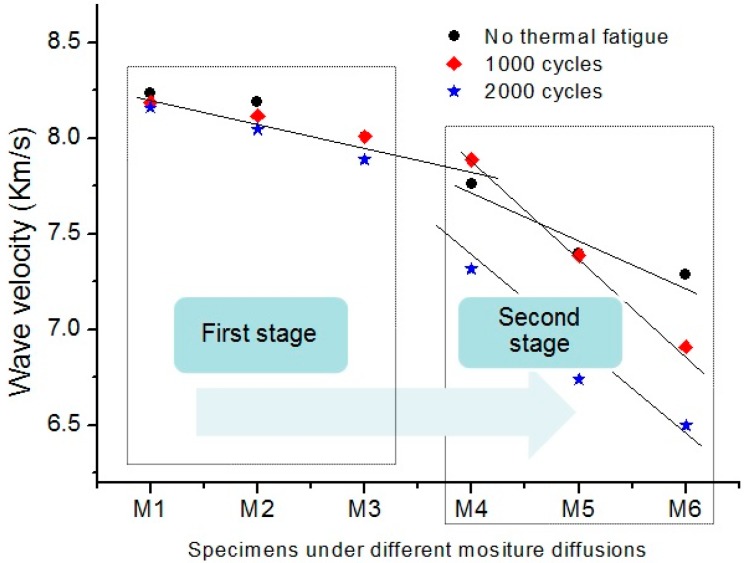
Variation of change rate of wave velocity in the different specimens subjected moisture diffusion.

With the increase of moisture diffusion, the change of the wave velocity decrease rate in this specimen that only subjected moisture diffusion is negligible or at least not as noticeable as that of specimens subjected multi-damages with both moisture diffusion and thermal fatigue, which means that the progressive of degradation in specimen is not as serious as that with multi-damages. As shown in Figure 9, all measured Lamb wave velocity variations in different specimens are plotted together to display only the relative change. Two stages can be clearly identified. In the first stage, the decrease rates of wave velocity in these specimens subjected to different thermal fatigue are almost constant, and no sharply variation of Lamb wave velocity. The degradation evolution of composite laminates can be characterized as the same ratio in this stage. And the influences of thermal fatigue on wave velocity variation is negligible. Moisture diffusion plays the dominate role for degrading the composite laminates in this stage. In the second stage, the significant variation of wave velocity represents the rapid degradation progressive of composite laminates. The significant differences among the specimens subjected to different thermal fatigue cycles is illustrated in this stage. The appearances of micro-defects induced by thermal fatigue accelerate the water absorption in composite laminates, and thus, accelerate the degradation procedure. The degradation evolution is attributed to both the thermal fatigue and moisture diffusion in composite laminates. The observation shows that there are two stages for damage initiation and progressive in the condition of multi-damages of thermal fatigue and moisture diffusion for aircraft composites suffered in-service aging.

The observation of two rates of decrease in Lamb wave velocity with the quantity of water absorption suggests presence of two stages of degradation evolution for in-service aircraft composite laminates. It is known that velocimetric methods which track changes in ultrasonic velocities due to variations in material properties can be used reliably for damage detection [19,26]. As shown in the Figure 9, the variation of decrease rate of Lamb wave velocity in the specimens is attributed to differences of degradation progressive stages. During degradation evolution, thermal fatigue plays a dominant role, resulting in the micro-damages, such as transverse matrix cracks, debonding and delaminations, while the debondings and matrix cracks could cause moisture diffusion along the fiber direction because of the filling of voids at the laminate interface. Meanwhile, moisture absorption of composite laminates can damage the interface over time. Water could interrupt the hydrogen bonding between the matrix and fiber, thereby weakening the laminate interface, and eventually accelerate the degradation evolution of composite laminates.

**Figure 10:**
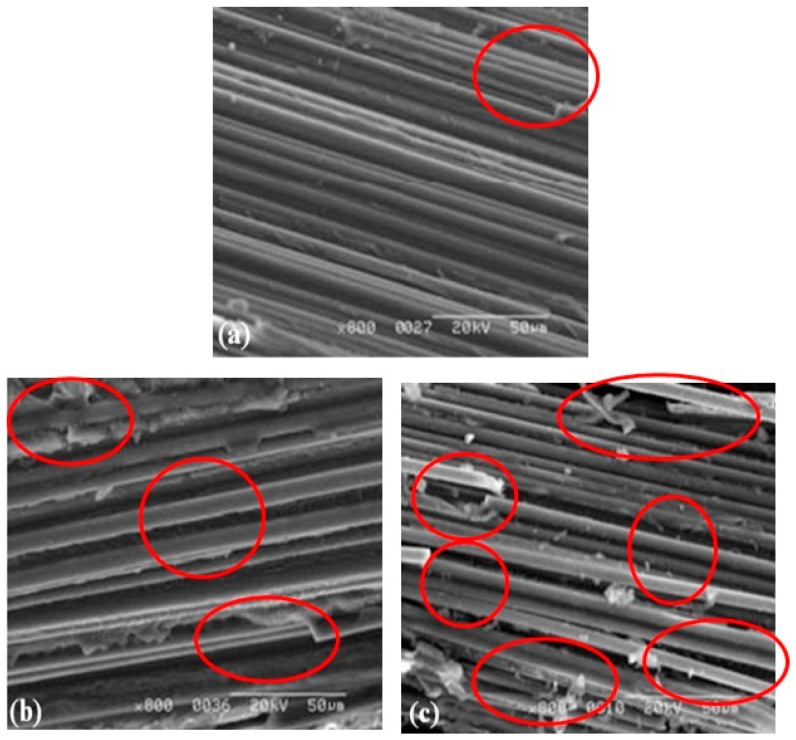
Observation of SEM micrographs of microstructural evolution in specimen (**a**) without thermal fatigue; (**b**) 1000 cycles thermal fatigue and (**c**) 2000 cycles thermal fatigue.

For further confirmation of the micro-structural evolution, scanning electron microscope (SEM) images are shown in Figure 10. The SEM images of specimens show the apparent increase of delaminations and fiber/matrix debondings caused by thermal fatigue damage. The debondings and matrix cracks could cause moisture diffusion along the fiber direction for the reason that the filling of voids at the laminate interface. Meanwhile, moisture absorption of composite laminates can damage the interface over time. Water could interrupt the hydrogen bonding between the matrix and fiber, thereby weakening the laminate interface, and eventually accelerate the degradation evolution of composite laminates.

With the improvement of composite materials manufacturing and bonding techniques, the nondestructive monitoring of degradation progressive can become an integral part of the structural material itself. The proposed technique in current investigation can be considered to be used in a structure-integrated damage monitoring system. Lamb waves can propagate over long distances without the need to scan point by point. So, it can be used to inspect large structures with high cost/time-efficiency. Inaccessible and hidden area of the structure can also be examined by this Lamb wave-based characterization technique. These advantages are quite attractive to increase the number of inspection intervals without increasing inspection time and cost. Another feature of proposed approach in this work is the simplicity of signal analysis. This advantage enhances computation speed to process the sensor signals in a reasonable time.

## 5. Conclusions

This paper aims to characterize the degradation evolution of unidirectional carbon/epoxy composite laminates by Lamb waves. Specimens were subjected to artificial thermal fatigue and moisture absorption to simulate the effect of temperature variation and excessive humidity of in-service environment. The experimental observation of the correlation between the change of Lamb wave velocity and various aging periods in specimens verifies that multi-damages can seriously affect the material properties of the composite laminates. Decrease rates of Lamb wave velocity in the specimen with different damage states indicate that two stages of progressive degradation due to the coupling damages mechanism for the interaction of moisture and thermal fatigue in composites. Moreover, as indicated in experimental results, the variations of Lamb wave velocity in degraded composites at first stage are not that significantly. Even velocimetric methods are reliable approaches for tracking the material degradation evolution. However, the use of these linear Lamb wave features is less sensitive at characterizing the initial stage of degradation, as indicated in the experimental test. The use of nonlinear Lamb wave features with appropriate mode selection and frequency turning is considered to monitor degradation progression in an early stage.

## Figures and Tables

**Table 1:** Density and elastic stiffness coefficients of carbon/epoxy composites.

ρ (kg/m^3^)	C_11_ (GPa)	C_12_ (GPa)	C_13_ (GPa)	C_22_ (GPa)	C_23_ (GPa)	C_33_ (GPa)	C_44_ (GPa)	C_55_ (GPa)	C_66_ (GPa)
1.5	146	5.81	5.81	11.07	6	11.07	2.55	3.82	4

**Table 2:** Specimens subjected to different thermal fatigue and moisture diffusions.

Thermal Fatigue Cycles	Moisture Percentages (%)					
	M1	M2	M3	M4	M5	M6
0 cycles	0	0.18	0.27	0.43	0.57	0.65
1000 cycles	0	0.19	0.31	0.49	0.59	0.7
2000 cycles	0	0.25	0.37	0.48	0.61	0.76
